# The contradictive findings between ultrasound, hysteroscopy and cytokines in women with nonhormonal IUDs suffering from menorrhagia: a prospective study

**DOI:** 10.1007/s00404-024-07457-7

**Published:** 2024-03-16

**Authors:** Hadel Watad, Udi Ifrach, David Stockheim, Vered Yulzari, Orly C. Meron, Miri Blank, Benjamin Sredni, Boaz Weisz, Shlomo B. Cohen

**Affiliations:** 1 Department of Obstetrics and Gynecology, Chaim Sheba Medical Center, Tel-Hashomer, Israel; 2 School of Medicine, Tel Aviv University, Tel Aviv, Israel; 3 Bar-Ilan University, Ramat Gan, Israel; 4 The Interdisciplinary Department of Social Sciences, Bar-Ilan University, Ramat Gan, Israel; 5 Zabludowicz Center for Autoimmune Diseases, Sheba Medical Center, Affiliated to the Faculty of Medicine, Tel Aviv University, Tel Aviv, Israel; 6 C.A.I.R. Institute, The Safdié AIDS and Immunology Research Center, The Mina & Everard Goodman Faculty of Life Sciences, Bar-Ilan University, Ramat Gan, Israel

**Keywords:** Contraception, Embedding, Inflammatory, Intermenstrual bleeding

## Abstract

**Purpose:**

The objective of this study is to assess the correlation between bleeding irregularities and the accurate placement of the intrauterine device (IUD) device in the uterine cavity, determined through transvaginal ultrasonography and hysteroscopy. In addition, the study aims to examine the cytokine profile in the uterine cavity and serum of patients experiencing bleeding irregularities after the insertion of nonhormonal IUDs.

**Methods:**

A prospective cohort study was conducted at a single tertiary medical center, wherein patients experiencing intermenstrual bleeding and spotting after the insertion of nonhormonal IUDs were enrolled. The study involved hysteroscopic and sonographic assessments of the uterine cavity and IUD placement, along with the analysis of blood and uterine cavity cytokine profiles.

**Results:**

During the period between July 2019 and February 2020, a total of eight patients who experienced intermenstrual bleeding and spotting after the insertion of nonhormonal IUDs were enrolled the study. One case was excluded since a progestative device was detected by ultrasound. Out of the five cases that underwent a thorough ultrasonographic assessment, three cases (60%) showed an embedded IUD. However, these findings were excluded by the hysteroscopic evaluation.

**Conclusion:**

The results suggest that ultrasonographic assessment may lead to an overdiagnosis of IUD mispositioning compared to hysteroscopy. In addition, both ultrasound and hysteroscopy have limitations in diagnosing the cause of bleeding in most cases. The role of local reactive inflammatory cytokines should be further studied.

## What does this study add to the clinical work

**Table:** 

Our study reveals that there is overdiagnosis of Intra-uterine device positioning when using ultrasonographic assessment for bleeding irregularities in comparison to hysteroscopy. Furthermore, in the majority of cases, both ultrasound and hysteroscopy do not provide sufficient value in determining the cause of bleeding.	

## Introduction

Bleeding irregularities associated with nonhormonal intrauterine devices (IUDs) can manifest as an increase in menstrual blood loss, prolonged bleeding during periods, or intermenstrual bleeding and spotting [1]. Several factors, such as the type of device, its surface area, duration of usage, individual response to the IUD, and parity, may influence the frequency and pattern of bleeding [1]; in addition, the cultural and social background of the woman may also play a role in her acceptance and motivation for IUD removal due to bleeding irregularities.

The pathogenesis of bleeding disturbances in IUD users is multifactorial and different etiologies have been suggested for different types of bleeding disturbances. The distortion of the endometrial vasculature by the presence of IUD leading to interstitial hemorrhage with the release of blood in an irregular pattern to the uterine cavity is the main pathogenesis that explains bleeding irregularities. The defective hemostatic mechanism in the IUD-exposed endometrium (Local increase in fibrinolytic activity) also contributes to the bleeding [1].

Ultrasonography is commonly used in the evaluation of the IUD placement as the arms and the stem of the copper IUD are echogenic. Ultrasonographic imaging has been accepted as the initial step in evaluation and management of malpositioned IUDs [2, 3], although some of the side effects occurred even when the IUD was in a normal position [4–6].

The aim of this study is to investigate possible etiologies of bleeding irregularities in nonhormonal IUD users and to evaluate the accuracy of ultrasonographic assessment of malposition of IUD device within the cavity as compared to hysteroscopic evaluation. We also aimed to examine the cytokine profile of serum and uterine fluids in such cases to assess the local inflammation process role.

## Materials and methods

A prospective cohort study of women suffering of bleeding irregularities following nonhormonal IUD insertion between July 2019 and February 2020, recruited via social media publications. We initially planned a clinical trial including study and control group; however, it was difficult to continue recruitment of participants since COVID 19 (Coronavirus) outbreak.

We included women with nonhormonal IUD inserted at least 3 months prior to recruitment, women with hormonal IUD, at menopause or peri-menopause and breastfeeding women were excluded. We also excluded women with bleeding that starts 5 days or less before expected menstruation date. Intermenstrual bleeding was defined as bleeding that starts after at least 1 day of cessation of period bleeding.

Eligible participants received a questionnaire and were asked to provide information regarding demographics, medical and surgical history, medication, obstetric and gynecological history and information regarding IUD insertion and side effects. Information regarding IUD included IUD type, duration of IUD use, previous ultrasonographic assessment following insertion, timing of intermenstrual bleeding following caseation of period bleeding, duration and amount of intermenstrual bleeding, accompanying side effects such as abdominal cramping, and previous intermenstrual bleeding in cases of previously used.

**Fig. 1:**
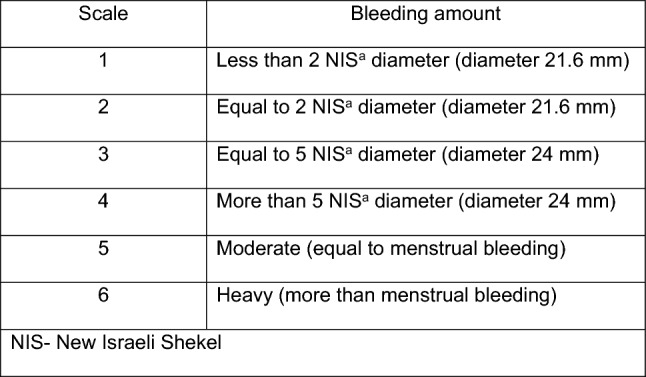
Vaginal bleeding amount quantification scale

Amount of bleeding was quantified according to our local coins, which is compatible with the scale described in Fig. 1.

Women were asked to rate their quality of life in scale from 0 to 10 (0 no disturbance, 10-significant disturbance to daily time quality). Following that, women had completed blood tests for hormonal profile, including progesterone and estradiol levels, blood tests for cytokine profile. All women were asked to complete transvaginal ultrasonographic assessment that included uterine position and dimensions, endometrial thickness, IUD displacement, distance IUD tip and body from myometrium, IUD embedding, uterine polyp, cesarean section niche and other abnormal findings including uterine fibroids. We used two and three-dimensional ultrasound (2D and 3D) using the Voluson E10 ultrasound system. All participants completed diagnostic hysteroscopy which included assessment of cervical lesions, IUD malposition, expulsion, embedding or perforation, disproportion between IUD and uterine dimensions, ulcerations and active bleeding, uterine malformations, fibroids and polyps, presence of endometrial inflammatory lesions or hyperplasia. We used a 2.9-mm TROPHYscope Campo hysteroscope (KARL STORZ, Tuttlingen, Germany) and 2 or 2.9 mm BETTOCCHI hysteroscope (KARL STORZ, Tuttlingen, Germany). Hysteroscopic access to the cervix was performed under vaginoscopy direct vision and did not require the use of a speculum. At first saline irrigated uterine fluid was obtained for cytokine profile assessment (2–3 mL), then hysteroscopy was continued using slowly flowing irrigating saline in aim to prevent IUD movement during the procedure. Hysteroscopy and ultrasound were done within 1–2 days of bleeding onset.

Serum and uterine fluids cytokine profile analysis which included levels of TNFα, IL-1β, IL-6 and IL-17A, The Luminex’s xMAP Cytokine “Multi-plex” array analysis was used.

The study was conducted at Sheba Medical Center, Tel-Hashomer, Israel; and was approved by the Sheba Medical Center institutional review board (5170-18). All participants provided informed consent.

### Patient and public involvement

Patients or the public were not involved in the design, or conduct, reporting, or dissemination plans of our research.

## Statistical analysis

Normally distributed continuous data were described using mean and standard deviation (SD), while non‐normally distributed data were described using median and interquartile range (IQR). All analyses were conducted using SPSS 22 (SPSS Inc., Chicago, IL, USA). Comparison tests were not performed. It is important to highlight that, given the absence of a control group, a comparative analysis was not conducted.

## Results

Eight eligible women who were recruited, one woman was excluded following ultrasonographic assessment that revealed a hormonal IUD. Baseline characteristics of the study groups are shown in Table 1.

**Table 1:** Demographic characteristics

*n* = 7	
Age, years (mean ± SD)	35.3 (5.6)
BMI^a^, kg/m^2^ (mean ± SD)	27.2 (5.1)
Gravidity (median, IQR)	4 (4–4.5)
Parity (median, IQR)	4 (4–4.5)
Abortions (median, IQR)	0 (0–0)

^a^*BMI* body mass index

All women had regular menses before IUD insertion, mean of menstrual cycle length was 28.9 (SD ± 0.97) days and lasted 5.8 (SD ± 0.78) days on average. Intermenstrual bleeding started 4.14 (SD ± 2.4) days on average and lasted 1.41 (SD ± 0.53) days on average. According to progesterone levels all tests were completed during the follicular stage. Six women out of 7 (85.7%) reported ultrasonographic assessment following IUD insertion that conformed right placement.

Baseline characteristics, IUD parameters including type and duration of use, menstrual and intermenstrual bleeding parameters are presented in Table 2. It is apparent that all women had regular menses before IUD insertion and that intermenstrual bleeding started 1–7 days following the end of period bleeding. Five cases reported onset of bleeding irregularities in conjunction with IUD insertion.

**Table 2:** Baseline characteristics and Intra-uterine device and bleeding data

Serial number	IUD^a^ type	Durations of IUD use (months)	Ultrasonographic assessment for IUD placement conformation following IUD insertion	Duration of intermenstrual irregularities (months)	Interval between last day menstruation and intermenstrual bleeding onset (days)	Duration of intermenstrual bleeding (days)	Bleeding amount scale	Disturbance of daily activity (0–10)
1	Ballerine IUD^a^	8	Normal placement	8	1	1	4	10
2	Copper IUD^a^ (not specified)	18	Normal placement	18	6	1.5	4	4
3	Mona Lisa IUD^a^	6	Normal placement	6	7	1	3	8
4	Gynefix IUD	24	Normal placement	12	7	2.5	3	8
5	Novo-t	12	Normal placement	12	1.5	1.5	1	8
6	Novo-t	12	N/A	12	2		4	
7	Ballerine IUD^a^	26	Normal placement	20	4.5	1	3	9

Disturbance of daily activity (0—no disturbance, 10—significant disturbance to daily time quality)

^a^*IUD* intrauterine device

Table 3 demonstrates result of ultrasonographic and hysteroscopic evaluation, it’s apparent that there is no consent between ultrasound and hysteroscopy concerning IUD displacement. At the day of recruitment, ultrasonographic assessment revealed embedding of the IUD in the uterine wall in three cases that was refuted following hysteroscopic assessment, moreover in two cases with IUD embedding as revealed by hysteroscopy, sonographic assessment didn’t reveal that.

**Table 3:** Sonographic and hysteroscopic findings

Serial number	Hysteroscopic findings	US findings	Uterine position	Uterus length (mm)	Uterus antero-posterior length (mm)	Uterus width (mm)	Endometrial thickness (mm)
1	Embedded IUD^a^	N/A	N/A	N/A	N/A	N/A	N/A
2	N/A	Embedded IUD^a^	Anteroflexed	71	46	53	6.7
3	N/A	Embedded IUD^a^	Anteroflexed	63	43	53	8.5
4	N/A	Embedded IUD^a^	Anteroflexed	63	28	57	11
5	Adenomyosis		Anteroflexed	57	37	49	6.8
6	Embedded IUD^a^, CS^b^ niche and uni-cornuate uterus	IUD^a^ displacement and CS^b^ niche	Central	54	34	60	3
7	N/A	N/A	Anteroflexed	70	40	46	12

^a^*IUD* intrauterine device

^*b*^*CS* cesarean section

Table 4 describes cytokine profile analysis; we aimed to estimate the contribution of local and systemic inflammatory processes to the onset of bleeding in the presence of the IUD, for technical issues, full analysis was conducted for 3 out of 7 cases. In two cases, the levels of inflammatory cytokines originated from dendritic and mononuclear cells from M1-type (TNFα, IL-1β IL-6) were in the normal range, which indicates that bleeding irregularities cannot be explained by the presence of systemic or local inflammation. However, the serum and uterine fluid levels of the inflammatory cytokines were elevated in the patient with uterine niche. Levels of IL-17, which is originated from lymphocyte T-helper 17 cells were in normal range in all cases.

**Table 4:** Cytokine profile

TNFa-uterine-pg/mL	IL-1B-uterine-pg/mL	IL-6-uterine-pg/mL	IL-17A-uterine-pg/mL	TNFa-serum-pg/mL	IL-1B-serum-pg/mL	IL-6-serum-pg/mL	IL-17A-serum-pg/mL
< 1.09↓	9.14	2.85	< 2.08↓	< 1.90↓	< 0.98↓	< 0.72↓	< 1.58↓
18.47	464.63	21.88	< 2.08↓	3.12	6	1.55	< 1.58↓
< 1.09↓	9.71	9.9	< 2.08↓	2.39	< 0.98↓	< 0.72↓	< 1.58↓

## Discussion

In the current study, poor correlation was found between ultrasonographic and hysteroscopic findings in women with IUD who suffer from bleeding irregularities. Ultrasound evaluation revealed IUD embedding that was not confirmed during hysteroscopy and vice versa. Although hysteroscopy is considered the gold standard for diagnosis of bleeding irregularities, according to the best of our knowledge, hysteroscopy was not previously used for evaluation of bleeding irregularities in women carrying a nonhormonal IUD. Another important but not surprising finding was that none of the cases in our study had anatomic aberrations that could be attributed to IUD insertion.

A cross-sectional survey published by Carugno et al. [7], showed that hysteroscopic procedures performed for IUD removal were mostly because of retention or fragmentation without mentioning of embedding, which strengthen our findings.

Previous studies evaluated uterine dimensions and Doppler in relation of IUD complications, however, most of the publications investigated menorrhagia without addressing other bleeding irregularities [3, 5], and conflicting results had been shown in the minority of studies which addressed bleeding irregularities including spotting [8, 9], which makes it difficult to compare our results with the previous results.

Endometrial inflammation occurring after copper IUD insertion is the main method of its action as contraception method, which prevents embryo implantation [10], multiple studies have addressed the inflammatory effect of IUD’s in animals and humans [11, 12]. Feng X et al. [13] had shown a correlation between VEGF levels and bleeding irregularities in vitro, however, to our knowledge, none of the works investigated the relationship between bleeding irregularities and the inflammatory response in vivo.

Primary results of cytokine profile analysis indicate that IUD associated bleeding irregularities are not mediated by systemic and/or local inflammatory reaction, and cases with niche may be associated with inflammatory reaction mediated by dendritic and mononuclear cells with T-cell lymphocyte involvement. However, those results should be investigated and addressed in further larger studies.

Our study is innovative, addressing bleeding irregularities in nonhormonal IUD users, while incorporating a novel approach by combining ultrasonographic and hysteroscopic evaluations with cytokine profile analysis, offering a new perspective in understanding the complex etiologies behind IUD-related bleeding irregularities. Moreover, we present detailed data about the study participants.

Our study has its own limitations, the small study group, the lack of control group, and the fact that cytokine profile analysis was not available in all cases. However, we should notify that COVID 19 pandemic limited our recruitment ability of study and control groups.

The limitations associated with the small sample size and the absence of a control group are acknowledged and recognized as crucial factors impacting the study’s generalizability. The decision to employ a small sample size was a result of logistical constraints; and we acknowledge that it may affect the external validity of our findings. Moreover, the absence of a control group poses challenges in establishing causality and isolating the specific effects related to the intervention under investigation. The unexpected challenges posed by the COVID-19 pandemic had an undeniable influence on the study’s methodology and participant recruitment.

The incomplete cytokine profile analysis due to technical issues, is a critical factor directly affecting the capacity to draw conclusive insights regarding the role of cytokines in IUD-related bleeding, a more comprehensive exploration is needed to elucidate its potential implication of cytokines on bleeding irregularities in such cases.

This study is a preliminary study that uses hysteroscopy for thorough assessment of bleeding irregularities in women carrying non hormonal IUD and compares the results with ultrasonographic assessment. For our knowledge, there is no previous publication that addressed those findings. Moreover, hysteroscopic evaluation was done adjacent to bleeding event which, according to our experience, Increases the chance of detecting a pathology.

Findings of our study should give patients a sense of security since no serious nor irreversible damage had been documented findings were found including in a careful assessment of uterine cavity.

Preliminary findings of our study need further research, and the cost-effectiveness of hysteroscopic and ultrasonographic evaluation should be addressed.

The study was approved at the Institutional review board number: 5170–18-SMC, April 3rd, 2019.

## Data Availability

The authors confirm that the data supporting the findings of this study are available within the article.

## References

[1] El-badrawi HH, Hafez ES (1980). IUD-induced uterine bleeding. Contracept Deliv Syst.

[2] Peri N, Graham D, Levine D (2007). Imaging of intrauterine contraceptive devices. J Ultrasound Med.

[3] Fadiloglu S (2018). Relationship between copper IUD complications and ultrasonographic findings. Arch Gynecol Obstet.

[4] Coskun E (2011). Effect of copper intrauterine device on the cyclooxygenase and inducible nitric oxide synthase expression in the luteal phase endometrium. Contraception.

[5] Mutlu I, Demir A, Mutlu MF (2014). Can uterine artery Doppler parameters predict copper intrauterine device-induced side effects?. Eur J Contracept Reprod Health Care.

[6] Faúndes D (2000). T-shaped IUDs accommodate in their position during the first 3 months after insertion. Contraception.

[7] Vitale SG (2022). In-office hysteroscopic removal of retained or fragmented intrauterine device without anesthesia: a cross-sectional analysis of an international survey. Updates Surg.

[8] Kaislasuo J (2015). Menstrual characteristics and ultrasonographic uterine cavity measurements predict bleeding and pain in nulligravid women using intrauterine contraception. Hum Reprod.

[9] Faúndes D (1997). No relationship between the IUD position evaluated by ultrasound and complaints of bleeding and pain. Contraception.

[10] Chou CH (2015). Divergent endometrial inflammatory cytokine expression at peri-implantation period and after the stimulation by copper intrauterine device. Sci Rep.

[11] Kim CR (2016). Immunologic evaluation of the endometrium with a levonorgestrel intrauterine device in solid organ transplant women and healthy controls. Contraception.

[12] Rivera Del Alamo MM (2021). Inflammatory markers in uterine lavage fluids of pregnant, non-pregnant, and intrauterine device implanted mares on days 10 and 15 post ovulation. Animals (Basel).

[13] Li L (2016). Analysis of the reason of abnormal uterine bleeding induced by copper corrosion of IUD Cu. Clin Exp Obstet Gynecol.

